# Study design of DIACORE (DIAbetes COhoRtE) – a cohort study of patients with diabetes mellitus type 2

**DOI:** 10.1186/1471-2350-14-25

**Published:** 2013-02-14

**Authors:** Lena Dörhöfer, Alexander Lammert, Vera Krane, Mathias Gorski, Bernhard Banas, Christoph Wanner, Bernhard K Krämer, Iris M Heid, Carsten A Böger

**Affiliations:** 1 Department of Internal Medicine II, Nephrology, University Hospital Regensburg, Franz-Josef-Strauß-Allee 11, 93042, Regensburg, Germany; 2 Department of Medicine, Nephrology, Endocrinology, Diabetology, Rheumatology; Universitätsmedizin Mannheim, Medical Faculty Mannheim, Heidelberg University, Theodor Kutzer Ufer 1-3, 68167, Mannheim, Germany; 3 Department of Internal Medicine I, Division of Nephrology, University Hospital Würzburg, Oberdürrbacherstraße 6, 97080, Würzburg, Germany; 4 Genetic Epidemiology, Department of Epidemiology and Preventive Medicine, University Regensburg, Franz-Josef-Strauß-Allee 11, 93042, Regensburg, Germany; 5 Institute of Genetic Epidemiology, Helmholtz Zentrum München, German Research Center for Environmental Health, Ingolstädter Landstr. 1, 85764, Neuherberg, Germany

**Keywords:** Diabetes mellitus type 2, Diabetic nephropathy, Chronic kidney disease, End stage renal disease, Cardiovascular morbidity, diabetes complications, Epidemiology, Genetics

## Abstract

**Background:**

Diabetes mellitus type 2 (DM2) is highly associated with increased risk for chronic kidney disease (CKD), end stage renal disease (ESRD) and cardiovascular morbidity. Epidemiological and genetic studies generate hypotheses for innovative strategies in DM2 management by unravelling novel mechanisms of diabetes complications, which is essential for future intervention trials. We have thus initiated the DIAbetes COhoRtE study (DIACORE).

**Methods:**

DIACORE is a prospective cohort study aiming to recruit 6000 patients of self-reported Caucasian ethnicity with prevalent DM2 for at least 10 years of follow-up. Study visits are performed in University-based recruiting clinics in Germany using standard operating procedures. All prevalent DM2 patients in outpatient clinics surrounding the recruiting centers are invited to participate. At baseline and at each 2-year follow-up examination, patients are subjected to a core phenotyping protocol. This includes a standardized online questionnaire and physical examination to determine incident micro- and macrovascular DM2 complications, malignancy and hospitalization, with a primary focus on renal events. Confirmatory outcome information is requested from patient records. Blood samples are obtained for a centrally analyzed standard laboratory panel and for biobanking of aliquots of serum, plasma, urine, mRNA and DNA for future scientific use. A subset of the cohort is subjected to extended phenotyping, e.g. sleep apnea screening, skin autofluorescence measurement, non-mydriatic retinal photography and non-invasive determination of arterial stiffness.

**Discussion:**

DIACORE will enable the prospective evaluation of factors involved in DM2 complication pathogenesis using high-throughput technologies in biosamples and genetic epidemiological studies.

## Background

Diabetes mellitus type 2 (DM2) is increasing in prevalence world wide, with more than 366 million people worldwide expected to be affected by the year 2030 [1]. DM2 interacts with other major risk factors such as hypertension and dyslipidemia, increasing the risk for micro- and macrovascular morbidity such as chronic kidney disease (CKD), end-stage renal disease (ESRD), atherosclerosis, coronary heart disease and cerebral ischemia [2-5]. For example, diabetic nephropathy is the underlying kidney disease of about every third patient starting renal replacement therapy [6-11].

In order to prevent cardio-renal complications among DM2 patients, it is necessary to understand the factors that influence their development. Hyperglycemia and hypertension are predominant factors. In the past 20 years, interventional studies have led to the development of effective strategies for improved blood pressure and glucose control [12-18], but nevertheless renal events such as microalbuminuria [12,14,19] and doubling of creatinine or ESRD [10,12,20] are only partially averted, with similar data for cardiovascular events.

This points towards other risk factors that add to the development of cardio-renal complications of DM2. Genetic factors appear to play an important role in renal complications, as evidenced by significant heritability of albuminuria and eGFR in diabetes patients [21]. Genetic variants associated with risk for type 2 diabetes, microalbuminuria and reduced kidney function have recently been identified [22-27], but most of the heritability observed in family studies is still unexplained [28,29]. Thus, additional genetic and non-genetic risk factors remain to be identified.

Key to the development of innovative strategies in DM2 management is the availability of high quality epidemiological study data.

In prospective general population-based studies [30,31], the number of persons developing DM2-associated cardio-renal events is limited. In contrast, a prospective cohort focusing on DM2 patients enables the evaluation of a broad range of DM2-specific risk factors for cardio-renal complications in an unbiased fashion and the collection of biosamples before and during the development of the events. Furthermore, such a patient group targets persons with an enhanced interest in participating in such a study and thus ensuring excellent compliance and high response during follow-up. However, large cohorts of DM2 patients with prospective biosample ascertainment are the exception [32-34].

Thus, we have initiated the DIAbetes COhoRtE study (DIACORE), a large cohort study for long term prospective follow-up with extensive biobanking. The aims of the DIACORE study are to discover novel mechanisms involved in the development and progression of diabetes complications, using modern high-throughput technologies for the unbiased analysis of biosamples.

Here, we report the study design.

## Methods

### Study design and main objective

DIACORE is a prospective cohort study aiming to elucidate mechanisms involved in the development and progression of DM2 complications using high-throughput technologies in collected biosamples (transcriptomics, proteomics and metabolomics), and genetic epidemiological studies including genome-wide association studies. Beginning 2/2010, the study aims to recruit 6000 patients with prevalent DM2 in a 4 year period. A standardized core phenotyping protocol including interview and medical examinations is performed at baseline and at every 2-year follow-up visit, with long-term follow-up of at least 10 years planned. Written informed consent is obtained after a standardized consent discussion at baseline. Blood samples are obtained from patients for a centrally analyzed standard laboratory panel and for biobanking of serum, plasma, urine, mRNA and DNA. Preferably, patients are fasting for at least eight hours at each visit. Patients are then subjected to a standardized physical examination and online questionnaire by specifically trained study nurses in the DIACORE examination rooms at the University based recruitment centers. In addition to the core phenotyping protocol, extended phenotyping is performed in a subset of patients (see below). All procedures are performed according to standard operating procedures. Adherence to these are ensured by regular training and internal audits. The DIACORE Study Group (see Appendix for current full member list) devises scientific analyses in the DIACORE study.

DIACORE is one of five cohort studies co-funded by the Kuratorium für Dialyse und Nierentransplantation (KfH) Foundation for Preventive Medicine e.V. The other cohorts are recruiting 5000 patients with CKD stage 3 or overt proteinuria to investigate CKD progression and associated complications [35], 2000 elderly individuals to establish a GFR estimation equation specific for the elderly [36], 625 children with CKD to investigate mechanisms involved in accelerated atherosclerosis [37] and 3000 patients after coronary angiography to investigate the relationship between coronary artery disease and CKD (CAD-REF registry). All 5 cohorts have harmonized their data protection standards (see below), the questionnaire and central laboratory parameters (http://www.kfh-stiftung-praeventivmedizin.de).

### Inclusion criteria

Inclusion criteria are the ability to provide written informed consent, age ≥18 years, self-reported Caucasian ethnicity and prevalent diabetes mellitus type 2. Diabetes mellitus type 2 is defined as need for blood glucose lowering medication, at least two measurements of fasting glucose ≥126 mg/dL or a 2-hour glucose value in OGTT >200 mg/dL [38-40]. Criteria for diagnosis of diabetes mellitus type 2 are obtained by self-report or from clinical records obtained from the patients’ health care provider.

### Exclusion criteria

Patients are excluded if, at baseline, they are on chronic renal replacement therapy (hemodialysis, peritoneal dialysis or transplantation), if, at baseline, there is a history of active malignancy (except those with basal cell carcinoma) within the last five years (prostatic cancer within the last two years), autoimmune disease potentially affecting kidney function (e.g. systemic lupus erythematodes), hemochromatosis, history of pancreoprivic or self-reported type 1 diabetes mellitus, acute infection or fever, pregnancy, chronic viral hepatitis or HIV-infection.

### Patient ascertainment

All DM2 patients aged at least 18 years living in the region around the study centers are invited to participate. To achieve this, the DIACORE study requests that local diabetologists, general practitioners and health insurance companies send written invitations, the study flyer and a stamped return post card to all DM2 patients in their records. Where permitted by the local diabetologist, DIACORE study personnel ascertains patients in the diabetologist’s office. Further, all DM2 patients without cancer (i.e. excluding patients with ICD-9 codes 140–239 or ICD-10 codes C00-C97) previously treated in each of the study center’s Departments of Internal Medicine aged at least 18 years and living in the region surrounding the recruiting center are invited by the local study center. All activities involving mailing of written invitations are flanked by supportive press releases. Patients are asked to reply by telephone or to use a stamped return post card in order to arrange an appointment with the study center for recruitment into DIACORE. Further, diabetologists and general practitioners are asked to distribute DIACORE study flyers to their patients in the doctor’s office.

### Core phenotyping protocol

#### Physical examination

The parameters of the physical examination are shown in Additional file 1: Table S1. Heart rate and blood pressure are assessed using a vital signs monitor after the patient has been seated for a five minute period. Three measurements are then performed every 2 minutes. The second and third measurements of blood pressure and the third measurement of pulse are recorded. Anthropometric parameters are measured in light clothing without shoes and after removal of any items from pockets. Weight is measured in kilograms with one decimal using a digital scale. Height is registered in centimeters standing face forward and shoeless, using a wall-mounted stadiometer. Waist and hip circumference are measured in centimeters using a 205 cm tape in a horizontal position by trained study nurses. The wearing of light clothing is recorded for adjustment in the online questionnaire [41]. The waist measuring point is defined as the smallest circumference between the upper iliac crest and lower costal margin. If this cannot be determined, e.g. in obese patients, the measurement is taken at the midway point between the upper iliac crest and lower costal margin. Hip circumference is measured at the largest circumference below the iliac crest over the trochanter major and over the buttocks.

#### Online questionnaire

After the physical examination and the taking of blood samples, patients are administered an online standardized questionnaire by trained study nurses (electronic case report form, eCRF), which assesses ethnicity, medical insurance status and participation in disease management programs, physical activity, nicotine and alcohol consumption, nephrological and urological history, cardiovascular risk factors and complications, medication, hospitalization and cancer history (Table 1). Further, information is obtained on how the patient first heard of the DIACORE study.

**Table 1:** Core phenotyping in DIACORE

**Phenotype category**	**Item**	**Instrument for obtaining phenotype**
General	Current Medication	Q
	Ethnicity	Q*
	Health insurance	Q
	Disease management program	Q
	Fasting status	Q
	Birth weight	Q*
Anthropometry	Systolic and diastolic blood pressure	P
	Heart rate	P
	Height	P
	Weight	P
	Waist circumference	P
	Hip circumference	P
Risk factors	Diabetes duration	Q*
	Hypertension duration	Q*
	Smoking history	Q
	Lipid status	L
	Physical activity	Q
	Family history of kidney disease	Q
Morbidity	*CAD*	Q
	History of CAD (WHO Rose Angina Questionnaire)	Q
	Myocardial infarction	Q#
	CABG, PCI, heart valve surgery	Q#
	*PAD and carotid atherosclerosis*	
	History of PAD (Edinburgh Questionnaire)	Q
	Vascular surgery or percutaneous intervention in peripheral and carotid vessels	Q#
	Cerebral ischemia	Q#
	*Microangiopathy*	
	History of kidney disease and biopsy results	Q#
	Kidney function parameters (e.g. eGFR, annual eGFR decline, albuminuria, change in albuminuria over time, dipstick urine proteinuria)	L
	Time to doubling of serum creatinine	L§
	Time to incident renal replacement therapy	Q#§
	Diabetic retinopathy requiring laser therapy	Q#
	*History of malignancy*	Q#
	*Hospitalisation*	Q#§
	*Mortality including cause*	Q#§

*CAD*: Coronary artery disease. *PAD*: peripheral artery disease. *Q*: self-reported information obtained by questionnaire. *L*: obtained by laboratory analysis of biosamples. *P*: obtained by physical examination.

* item determined at baseline visit only.

# items validated by obtaining medical records.

§ item determined at follow-up visits only.

#### Biosampling

Whole blood samples are drawn after the patients have rested in a seated position for at least 15 minutes, applying mild venous stasis and using a 21G butterfly needle (Sarstedt), into serum gel, EDTA, sodium fluoride (all Sarstedt, Germany) and Paxgene® tubes (PreAnalytix GmbH, Switzerland). At the end of the interview, the patient is asked to provide a spot midstream urine sample into a sterile 100 mL cup (Sarstedt, Germany). A urine dipstick (Combur, Germany) is performed in the fresh urine sample within 15 minutes after donation for quality assurance (erythrocyturia, leukocyturia and nitrite) and for semiquantitative determination of proteinuria.

Blood and urine sample processing follows identical standard operating procedures in all recruiting centers: All samples are kept at room temperature for 30 minutes, then all blood and urine tubes except the EDTA and sodium fluoride tubes for whole blood count, HbA1c, DNA isolation and blood glucose determination are centrifuged at 2500 G for 10 minutes, after which a 2mL urine sample is transferred into a sterile polyethylene tube, with subsequent storage of all samples at 4°C until further processing. A centrifuged serum gel tube, an EDTA anticoagulated whole blood tube, the sodium fluoride anticoagulated blood tube and the 2 mL urine sample are packaged for delivery to the central laboratory for determination of a standard clinical chemistry panel while a 500 μL serum sample is frozen at −20°C until batch transport to the central laboratory for measurement of insulin activity (Additional file 1: Table S2). After 2–4 hours, all remaining serum and urine, and 2mL EDTA-plasma are aliquoted into 2D barcoded polypropylene tubes for long-term storage in −80°C freezers at each of the recruiting centers with electronic temperature monitoring. The archiving of the 2D barcoded tubes is done within the eCRF using an integrated software module (BioArchive, MEDEORA GmbH; Köln, Germany). The Paxgene® tube is handled according to the instruction provided by the producer before being transferred for long term storage at −80°C. Uncentrifuged EDTA-anticoagulated whole blood is stored at −80°C until DNA isolation, which is performed centrally at the University of Regensburg using a Qiagen kit.

#### Clinical chemistry panel

A clinical chemistry panel is obtained from every patient at every visit in a central laboratory using state of the art analyzers (Additional file 1: Table S2). Blood samples are transported weekdays at a temperature range of 4-8°C from recruiting centers to the central laboratory, except for the 500 μL frozen serum sample which is sent in batches on dry ice. Surveillance of temperature during transport is performed at regular intervals.

#### Report of examination results to patients

Feedback to the patient is provided in the case of severely abnormal results (blood pressure > 180 mmHg systolic and 110 mmHg diastolic or < 90 mmHg systolic and 50 mmHg diastolic ; Pulse > 90/min and < 50 /min or irregular) at the end of the examination. Participants with abnormal results are either referred to the appropriate health care provider or, in medical emergencies, brought to the emergency room for further assessment.

A written report including results of the physical examination and of the standard laboratory panel is mailed to the patient who is asked to appropriately inform their primary health care provider as previously reported [36]. Each parameter is reported with age and gender specific reference values.

### Follow-up examination

Follow-up examinations are performed every two years with the same standard operating procedures applied as at baseline. At each of these examinations, the manifestation of phenotypes listed in Table 1 since the last examination is recorded, including their date of manifestation to determine the time of onset.

### Clinical outcomes

Clinical outcomes (Table 1, category “Morbidity”) are assessed at baseline (for cross-sectional analyses) and at each follow-up (for longitudinal analyses). Several phenotypes are defined by laboratory parameters (Additional file 1: Table S2). For example, kidney function is defined by estimating the GFR (eGFR) from a serum creatinine or cystatin C measurement [42-46] and the CKD status is derived from eGFR. Similarly, the degree of albuminuria is determined from the albumin-creatinine-ratio in the random spot urine.

The clinical end points are validated at every visit including the baseline visit by the DIACORE end point committee, consisting of two physicians. Medical documentation is requested up to three times from the patients’ treating physicians, and subsequently assessed independently by the members of the end point committee. If the verdict of the two members does not match, then agreement is sought by joint consultation. If a patient-reported end point cannot be confirmed by available documentation or if adequate medical documentation is not available, then that end point is coded as “not validated”. Thus, the validated end points form a subset of the overall set of self-reported end points.

### Extended phenotyping

DIACORE is open to ancillary studies to extend the current core phenotyping panel. Planned ancillary studies entail more intense phenotyping in a random subset of patients, e.g. screening for sleep apnea syndrome, non-mydriatic retinal photography, measurements of skin autofluorescence and arterial stiffness parameters.

### Power considerations

The calculation of DIACORE’s planned sample size of 6000 DM2 patients is based on estimations for renal events made in the UKPDS study [47]: after approximately 10 years with DM2, 25% of patients had developed increased urinary albumin excretion (microalbuminuria), about 2-3% of patients progressed each year from normalbuminuria to macroalbuminuria and then reduced eGFR or ESRD, and annual mortality rate increased from 1.2 to 4.6% as patients progressed from normalbuminuria to macroalbuminuria. Loss to follow up was on average 1% per year [47].

Within the fifth year we expect to have recruited a cohort of 6000 participants. Based on the estimations made by Adler et al. [47] but assuming a more conservative drop out rate of 5% per year and a mortality rate of 4% per year, 750 (12.5%) participants will be lost to follow up and 600 (10%) will have died after the five years. We estimate that 1500 patients (25%) will have baseline microalbuminuria and that after 5 years 375 (6.25%) will develop microalbuminuria, macroalbuminuria, incident eGFR<60 ml/min/1.73 m^2^ or ESRD respectively, thus providing DIACORE with sufficient cases for cross-sectional and longitudinal analyses.

### Data handling and protection

DIACORE constitutes a human biomaterial databank with implications for protection of individual patient data. At baseline assessment every participant receives a pseudonym ID. Personal information such as name and address is stored separately. All information gathered from the online questionnaire is stored in an online electronic case report form (Sapphire, MEDEORA GmbH; Köln, Germany) under a second pseudonym ID which is transformed from the first pseudonym ID by a commercial online provider. Transmission of data is encrypted using a 256bit-SSL standard.

The data protection strategy is based on the generic model of the “platform for technology and methods for networked medical research” (TMF e.V., http://www.tmf-ev.de) supported by the German Ministry of Education and Research. A subset of the parameters obtained by questionnaire and by central laboratory testing is transferred to a central database which pools data from all five studies co-funded by the KfH Foundation for Preventive Medicine.

### Ethical approval and informed consent

The protocol, the data protection strategy and the study procedures have been approved by the Ethics Committees of participating institutions and are in accordance with the Declaration of Helsinki. Patients participate in DIACORE only after providing informed written consent.

## Discussion

The DIAbetes COhoRtE (DIACORE) study is a large cohort of prevalent DM2 patients in Germany that started recruitment in February 2010 for long term prospective observation with follow-up examinations every 2 years. DIACORE aims to elucidate novel mechanisms involved in the development of DM2 complications with a focus on diabetes-associated kidney disease. To achieve this, standardized phenotyping is being performed every 2 years, with biobanking of serum, plasma, DNA, mRNA and urine for future scientific analyses. All procedures are standardized for collaborative projects with population-based [30,31] and disease-based cohorts [35,36]. Due to its size and prospective design, DIACORE is an important extension to the current worldwide repertoire of DM2 cohorts of Caucasian descent investigating diabetes complications [15,48-51] and the first of comparable size in Germany.

DIACORE aims to recruit a representative sample of outpatient, ambulating DM2 patients in Germany. We have adopted a strategy of addressing eligible patients via several channels to achieve this aim: 1) a wide range of public communication media including local press and patient organisations, 2) major health insurance companies, 3) all general practitioners and 4) all diabetes specialists in a defined geographic region. Once the cohort is recruited, comparisons with the DM2 subsets of large population-based cohorts [4,5,52,53] and with data from German diabetes disease management data bases will serve to assess potential bias.

We have purposefully avoided using the term “diabetic nephropathy” to describe renal outcomes in DIACORE since a clinical diagnosis of diabetic nephropathy based only on proteinuria, eGFR and history is subject to misclassification if a kidney biopsy is not performed [54-57]: in biopsy studies, nondiabetic glomerulopathies could be detected in about 23-33% of DM2 patients with macroalbuminuria. Further, it has been shown in DM1 and DM2 patients that a substantial proportion of DM patients develop decreased eGFR without concurrent or prior elevation of albuminuria [58-62], suggesting an underlying non-diabetic kidney disease such as hypertension-associated nephrosclerosis. In addition, genetic studies have demonstrated disparate genetic underpinnings for albuminuria and eGFR in both the general population and in diabetes patients [21,25,27,63]. Taken together, these data support the concept of separate analyses of these traits.

DM2 incidence and prevalence is increasing in industrialized countries. In addition to the disease itself, its complications are a major public health and financial challenge. Thus, the investigation of factors involved in the development and progression of diabetic complications, including CKD, is essential to develop novel strategies for prevention and therapy.

An innovative approach for uncovering mechanisms underlying common diseases includes unbiased high-throughput screening of biomaterials using methods such as genome-wide association studies (GWAS) [29], and complementary analyses of the metabolome, transcriptome and proteome [64,65]. This approach has the advantage of a systematic analysis without prior biological hypothesis, thus bearing the potential of uncovering unexpected and completely novel mechanisms of disease. In this sense, GWAS have been successful for the majority of common diseases [66] including DM2 and chronic kidney disease (CKD). For example, *UMOD*, the gene encoding Uromodulin or Tamm-Horsfall-Protein, is among the loci associated with CKD [24,26,27,67,68]. This protein has long been known to be the quantitatively most important protein in human urine, but its function is unknown. Research is now focusing on the mechanisms involved in affecting this established association with risk for CKD in the general population [69].

In contrast, genetic variants associated with DM2-associated kidney disease have not consistently been replicated [28], possibly owing to lack of sufficient power either due to low sample size or to the small effects of genetic variants in common disease. Though DIACORE will be one of the largest DM2 cohorts of European descent, power may not be sufficient for detecting genetic variants with small effects sizes. However, since DIACORE’s study protocol is standardized for collaborative projects with other cohorts, joint meta-analysis is an excellent option for increasing power. Pooling data from several studies in international collaborations such as the CKDGen consortium has been successful in the past in analyzing the genetic underpinning of kidney phenotypes in the general population [24,25,27,68].

The strengths of the DIACORE study include its large size, prospective design, rigorous protocol standardization, centralized data management and excellent pre-analytics in biosample archiving. There are limitations that warrant discussion. As management of diabetes and its complications has improved significantly since publication of the study that our power calculation is based on [47], event rates in DIACORE may be lower than expected. However, the projected sample size makes DIACORE one of the largest DM2 cohorts world wide. Furthermore, bias may arise due to our recruitment strategy, but DM2 patients are addressed repeatedly along multiple channels in the same geographic regions thus providing broad feed back among the targeted population of ambulating DM2 patients. In addition, the questionnaire is designed to help quantify any recruitment bias. We cannot exclude or quantify a potential, small effect on outcomes caused by reporting the laboratory and physical examination results to patients. However, this reporting is an imperative medical duty to volunteering patients. Finally, urinary albumin excretion is estimated from one spot urine sample as it is logistically unfeasible to collect the recommended three urine samples. To compensate this drawback, dipstick testing on fresh urine is performed to exclude urinary tract infection, and the time at which urine is donated is recorded.

In summary, DIAbetes COhoRtE (DIACORE) is a cohort study with a biosample repository aiming to recruit 6000 prevalent diabetes mellitus type 2 patients of Caucasian descent in Germany for long term prospective observation with follow-up examinations every 2 years. Main aims are to discover novel mechanisms involved in developing kidney disease and cardiovascular morbidity among diabetes patients, using systematic genetic and biomarker analyses. In the long-term, DIACORE aims to provide impetus for the development of novel strategies in the prevention and treatment of DM2 complications.

## Appendix

### DIACORE study group

Lena Dörhöfer, Alexander Lammert, Konstantin Dumann, Britta Hörrmann, Sybille Hauske, Mathias Gorski, Michael Kirsch, Stephan W. Reinhold, Axel Andreae, Gerhard Haas, Sabine Haas, Jochen Manz, Johann Nusser, Günther Kreisel, Gerhard Bawidamann, Frederik Mader, Susanne Kißkalt, Johann Hartl, Thomas Segiet, Christiane Gleixner, Michael Arzt, Vera Krane, Andrej Wöhrmann (MEDEORA GmbH), Norbert Schmeisser (MEDEORA GmbH), Winfried März, Simone Neumeier, Sarah Hufnagel, Petra Jackermeier, Sabrina Obermüller, Philipp Pagel (Lipofit GmbH), Fritz Huber (Lipofit GmbH), Volker Pfahlert (Lipofit GmbH), Christian Scholz, Monika Schober (Chief of Supply Management, Allgemeine Ortskrankenkasse Bayern), Cornelia Heinrich (Communication Manager, Allgemeine Ortskrankenkasse Bayern), Thomas Bohnhoff (Disease Management, Techniker Krankenkasse), Thomas Heilmann (Head of Disease Management, Techniker Krankenkasse), Stefan Stern (Consulting Physician, Allgemeine Ortskrankenkasse Bayern), Andreas Utz (Head of Department, Allgemeine Ortskrankenkasse Bayern), Georg Zellner (Chief of Supply Management, Deutsche Angestellten Krankenkasse), Christoph Wanner, Bernhard Banas, Bernhard K. Krämer, Iris M. Heid, Carsten A. Böger.

## Competing interest

The authors declare that they have no competing conflicts of interest.

## Authors’ contributions

LD, IMH, CAB drafted the manuscript. LD, AL, BKK, CAB are involved in patient recruitment. LD, CAB, MG and IMH are responsible for statistical and power considerations. All authors are involved in study design and have critically reviewed and provided final approval of the manuscript.

## Authors’ information

For a full list of authors in the DIACORE Study Group, please see the Appendix.

## Pre-publication history

The pre-publication history for this paper can be accessed here:

http://www.biomedcentral.com/1471-2350/14/25/prepub

## Supplementary Material

Additional file 1: Table S1 Physical examination in the core phenotyping protocol. **Table S2.** Core laboratory parameters. Click here for file

## References

[B1] Wild S Roglic G Green A Sicree R King H Global prevalence of diabetes: estimates for the year 2000 and projections for 2030 Diabetes Care 2004 27 1047 1053 10.2337/diacare.27.5.1047 15111519

[B2] Gross JL de Azevedo MJ Silveiro SP Canani LH Caramori ML Zelmanovitz T Diabetic nephropathy: diagnosis, prevention, and treatment Diabetes Care 2005 28 164 176 10.2337/diacare.28.1.164 15616252

[B3] Anand SS Dagenais GR Mohan V Glucose levels are associated with cardiovascular disease and death in an international cohort of normal glycaemic and dysglycaemic men and women: the EpiDREAM cohort study Eur J Prev Cardiol 2012 19 755 764 10.1177/1741826711409327 21551215

[B4] Bash LD Astor BC Coresh J Risk of incident ESRD: a comprehensive look at cardiovascular risk factors and 17 years of follow-up in the Atherosclerosis Risk in Communities (ARIC) Study Am J Kidney Dis 2010 55 31 41 10.1053/j.ajkd.2009.09.006 19932544

[B5] Hardy DS Hoelscher DM Aragaki C Association of glycemic index and glycemic load with risk of incident coronary heart disease among Whites and African Americans with and without type 2 diabetes: the Atherosclerosis Risk in Communities study Ann Epidemiol 2010 20 610 616 10.1016/j.annepidem.2010.05.008 20609341 PMC3085981

[B6] Williams ME Diabetic CKD/ESRD 2010: a progress report? Semin Dial 2010 23 129 133 10.1111/j.1525-139X.2009.00698.x 20210917

[B7] Van Dijk PC Jager KJ Stengel B Gronhagen-Riska C Feest TG Briggs JD Renal replacement therapy for diabetic end-stage renal disease: data from 10 registries in Europe (1991–2000) Kidney Int 2005 67 1489 1499 10.1111/j.1523-1755.2005.00227.x 15780102

[B8] Foley RN Collins AJ End-stage renal disease in the United States: an update from the United States Renal Data System J Am Soc Nephrol 2007 18 2644 2648 10.1681/ASN.2007020220 17656472

[B9] Centers for Disease Control and Prevention (CDC) Incidence of end-stage renal disease attributed to diabetes among persons with diagnosed diabetes --- United States and Puerto Rico, 1996–2007 MMWR Morb Mortal Wkly Rep 2010 59 1361 1366 21030940

[B10] Keane WF Brenner BM de Zeeuw D The risk of developing end-stage renal disease in patients with type 2 diabetes and nephropathy: the RENAAL study Kidney Int 2003 63 1499 1507 10.1046/j.1523-1755.2003.00885.x 12631367

[B11] Icks A Haastert B Genz J Incidence of renal replacement therapy (RRT) in the diabetic compared with the non-diabetic population in a German region, 2002–08 Nephrol Dial Transplant 2011 26 264 269 10.1093/ndt/gfq398 20624774

[B12] Ismail-Beigi F Craven T Banerji MA Effect of intensive treatment of hyperglycaemia on microvascular outcomes in type 2 diabetes: an analysis of the ACCORD randomised trial Lancet 2010 376 419 430 10.1016/S0140-6736(10)60576-4 20594588 PMC4123233

[B13] Gerstein HC Miller ME Byington RP Effects of intensive glucose lowering in type 2 diabetes N Engl J Med 2008 358 2545 2559 18539917 10.1056/NEJMoa0802743 PMC4551392

[B14] Patel A MacMahon S Chalmers J Intensive blood glucose control and vascular outcomes in patients with type 2 diabetes N Engl J Med 2008 358 2560 2572 18539916 10.1056/NEJMoa0802987

[B15] Intensive blood-glucose control with sulphonylureas or insulin compared with conventional treatment and risk of complications in patients with type 2 diabetes (UKPDS 33). UK Prospective Diabetes Study (UKPDS) Group Lancet 1998 352 837 853 9742976

[B16] Tight blood pressure control and risk of macrovascular and microvascular complications in type 2 diabetes: UKPDS 38. UK Prospective Diabetes Study Group BMJ 1998 317 703 713 10.1136/bmj.317.7160.703 9732337 PMC28659

[B17] Haller H Ito S Izzo JL Jr Olmesartan for the delay or prevention of microalbuminuria in type 2 diabetes N Engl J Med 2011 364 907 917 10.1056/NEJMoa1007994 21388309

[B18] Reaven PD Moritz TE Schwenke DC Intensive glucose-lowering therapy reduces cardiovascular disease events in veterans affairs diabetes trial participants with lower calcified coronary atherosclerosis Diabetes 2009 58 2642 2648 10.2337/db09-0618 19651816 PMC2768182

[B19] Agrawal L Azad N Emanuele NV Observation on renal outcomes in the veterans affairs diabetes trial Diabetes Care 2011 34 2090 2094 10.2337/dc11-0175 21775749 PMC3161270

[B20] Lewis EJ Hunsicker LG Clarke WR Renoprotective effect of the angiotensin-receptor antagonist irbesartan in patients with nephropathy due to type 2 diabetes N Engl J Med 2001 345 851 860 10.1056/NEJMoa011303 11565517

[B21] Placha G Canani LH Warram JH Krolewski AS Evidence for different susceptibility genes for proteinuria and ESRD in type 2 diabetes Adv Chronic Kidney Dis 2005 12 155 169 10.1053/j.ackd.2005.02.002 15822051

[B22] Sladek R Rocheleau G Rung J A genome-wide association study identifies novel risk loci for type 2 diabetes Nature 2007 445 881 885 10.1038/nature05616 17293876

[B23] Wellcome Trust Case Control Consortium Genome-wide association study of 14,000 cases of seven common diseases and 3,000 shared controls Nature 2007 447 661 678 10.1038/nature05911 17554300 PMC2719288

[B24] Böger C Gorski M Li M Association of eGFR-related loci identified by GWAS with incident CKD and ESRD PLoS Genet 2011 7 1 8 10.1371/journal.pgen.1002292 PMC3183079 21980298

[B25] Böger CA Chen MH Tin A CUBN Is a Gene Locus for Albuminuria J Am Soc Nephrol 2011 22 555 570 10.1681/ASN.2010060598 21355061 PMC3060449

[B26] Köttgen A Glazer NL Dehghan A Multiple loci associated with indices of renal function and chronic kidney disease Nat Genet 2009 41 712 717 10.1038/ng.377 19430482 PMC3039280

[B27] Köttgen A Pattaro C Böger CA New loci associated with kidney function and chronic kidney disease Nat Genet 2010 42 376 384 10.1038/ng.568 20383146 PMC2997674

[B28] Böger C Heid I Chronic Kidney Disease: Novel Insights from Genome Wide Association Studies Kidney Blood Press Res 2011 34 27 36 10.1159/000326901 21691125

[B29] McCarthy MI Abecasis GR Cardon LR Genome-wide association studies for complex traits: consensus, uncertainty and challenges Nat Rev Genet 2008 9 356 369 10.1038/nrg2344 18398418

[B30] The National Cohort. A prospective epidemiologic study resource for health and disease research in Germany 2011 http://www.nationale-kohorte.de/content/wissenschaftliches_konzept_der_nationalen_kohorte.pdf. **Accessed March 26, 2012.**

[B31] Baumeister SE Böger CA Krämer BK Effect of chronic kidney disease and comorbid conditions on health care costs: A 10-year observational study in a general population Am J Nephrol 2010 31 222 229 10.1159/000272937 20068286

[B32] Almgren P Lehtovirta M Isomaa B Heritability and familiality of type 2 diabetes and related quantitative traits in the Botnia Study Diabetologia 2011 54 2811 2819 10.1007/s00125-011-2267-5 21826484

[B33] Pearson ER Donnelly LA Kimber C Variation in TCF7L2 influences therapeutic response to sulfonylureas: a GoDARTs study Diabetes 2007 56 2178 2182 10.2337/db07-0440 17519421

[B34] Igo RP Jr Iyengar SK Nicholas SB Genomewide linkage scan for diabetic renal failure and albuminuria: the FIND study Am J Nephrol 2011 33 381 389 10.1159/000326763 21454968 PMC3078269

[B35] Eckardt KU Barthlein B Baid-Agrawal S The German Chronic Kidney Disease (GCKD) study: design and methods Nephrol Dial Transplant 2011 27 1454 1460 21862458 10.1093/ndt/gfr456

[B36] Schaeffner ES van der Giet M Gaedeke J The Berlin initiative study: the methodology of exploring kidney function in the elderly by combining a longitudinal and cross-sectional approach Eur J Epidemiol 2010 25 203 210 10.1007/s10654-010-9424-x 20094758

[B37] Querfeld U Anarat A Bayazit AK The Cardiovascular Comorbidity in Children with Chronic Kidney Disease (4C) study: objectives, design, and methodology Clin J Am Soc Nephrol 2010 5 1642 1648 10.2215/CJN.08791209 20576824 PMC2974406

[B38] WHO 2011 http://www.who.int/diabetes/publications/report-hba1c_2011.pdf (Accessed on June 07, 2011

[B39] WHO 2011 http://whqlibdoc.who.int/publications/2006/9241594934_eng.pdf (Accessed on December 22, 2011)

[B40] American Diabetes Association Diagnosis and classification of diabetes mellitus Diabetes Care 2010 33 Suppl 1 S62 69 20042775 10.2337/dc10-S062 PMC2797383

[B41] Pischon T Boeing H Hoffmann K General and abdominal adiposity and risk of death in Europe N Engl J Med 2008 359 2105 2120 10.1056/NEJMoa0801891 19005195

[B42] Levey AS Bosch JP Lewis JB Greene T Rogers N Roth D A more accurate method to estimate glomerular filtration rate from serum creatinine: a new prediction equation. Modification of Diet in Renal Disease Study Group Ann Intern Med 1999 130 461 470 10075613 10.7326/0003-4819-130-6-199903160-00002

[B43] Levey AS Coresh J Greene T Using standardized serum creatinine values in the modification of diet in renal disease study equation for estimating glomerular filtration rate Ann Intern Med 2006 145 247 254 16908915 10.7326/0003-4819-145-4-200608150-00004

[B44] Levey AS Stevens LA Schmid CH A new equation to estimate glomerular filtration rate Ann Intern Med 2009 150 604 612 19414839 10.7326/0003-4819-150-9-200905050-00006 PMC2763564

[B45] Böger CA Kronenberg F How healthy are your vessels?–Check your urine! Atherosclerosis 2012 220 38 41 10.1016/j.atherosclerosis.2011.09.033 22018641

[B46] Stevens LA Coresh J Schmid CH Estimating GFR using serum cystatin C alone and in combination with serum creatinine: a pooled analysis of 3,418 individuals with CKD Am J Kidney Dis 2008 51 395 406 10.1053/j.ajkd.2007.11.018 18295055 PMC2390827

[B47] Adler AI Stevens RJ Manley SE Bilous RW Cull CA Holman RR Development and progression of nephropathy in type 2 diabetes: the United Kingdom Prospective Diabetes Study (UKPDS 64) Kidney Int 2003 63 225 232 10.1046/j.1523-1755.2003.00712.x 12472787

[B48] Palmer CN Kimber CH Doney AS Combined effect of inflammatory gene polymorphisms and the risk of ischemic stroke in a prospective cohort of subjects with type 2 diabetes: a Go-DARTS study Diabetes 2010 59 2945 2948 10.2337/db09-1690 20622166 PMC2963555

[B49] Ahluwalia TS Lindholm E Groop LC Common variants in CNDP1 and CNDP2, and risk of nephropathy in type 2 diabetes Diabetologia 2011 54 2295 2302 10.1007/s00125-011-2178-5 21573905

[B50] Mann JF Schmieder RE McQueen M Renal outcomes with telmisartan, ramipril, or both, in people at high vascular risk (the ONTARGET study): a multicentre, randomised, double-blind, controlled trial Lancet 2008 372 547 553 10.1016/S0140-6736(08)61236-2 18707986

[B51] The SYSKID Consortium http://www.syskid.eu. Accessed March 26, 2012

[B52] Preis SR Pencina MJ Hwang SJ Trends in cardiovascular disease risk factors in individuals with and without diabetes mellitus in the Framingham Heart Study Circulation 2009 120 212 220 10.1161/CIRCULATIONAHA.108.846519 19581493 PMC2789428

[B53] Fox CS Larson MG Leip EP Meigs JB Wilson PW Levy D Glycemic status and development of kidney disease: the Framingham Heart Study Diabetes Care 2005 28 2436 2440 10.2337/diacare.28.10.2436 16186276

[B54] Mazzucco G Bertani T Fortunato M Different patterns of renal damage in type 2 diabetes mellitus: a multicentric study on 393 biopsies Am J Kidney Dis 2002 39 713 720 10.1053/ajkd.2002.31988 11920336

[B55] Haider DG Peric S Friedl A Kidney biopsy in patients with diabetes mellitus Clin Nephrol 2011 76 180 185 21888854 10.5414/cn106955

[B56] Suzuki D Takano H Toyoda M Evaluation of renal biopsy samples of patients with diabetic nephropathy Intern Med 2001 40 1077 1084 10.2169/internalmedicine.40.1077 11757760

[B57] Parving HH Gall MA Skott P Prevalence and causes of albuminuria in non-insulin-dependent diabetic patients Kidney Int 1992 41 758 762 10.1038/ki.1992.118 1513098

[B58] Molitch ME Steffes M Sun W Development and progression of renal insufficiency with and without albuminuria in adults with type 1 diabetes in the diabetes control and complications trial and the epidemiology of diabetes interventions and complications study Diabetes Care 2010 33 1536 1543 10.2337/dc09-1098 20413518 PMC2890355

[B59] Retnakaran R Cull CA Thorne KI Adler AI Holman RR Risk factors for renal dysfunction in type 2 diabetes: U.K. Prospective Diabetes Study 74 Diabetes 2006 55 1832 1839 10.2337/db05-1620 16731850

[B60] Kramer HJ Nguyen QD Curhan G Hsu CY Renal insufficiency in the absence of albuminuria and retinopathy among adults with type 2 diabetes mellitus JAMA 2003 289 3273 3277 10.1001/jama.289.24.3273 12824208

[B61] Perkins BA Ficociello LH Ostrander BE Microalbuminuria and the risk for early progressive renal function decline in type 1 diabetes J Am Soc Nephrol 2007 18 1353 1361 10.1681/ASN.2006080872 17329575

[B62] Costacou T Ellis D Fried L Orchard TJ Sequence of progression of albuminuria and decreased GFR in persons with type 1 diabetes: a cohort study Am J Kidney Dis 2007 50 721 732 10.1053/j.ajkd.2007.08.005 17954285

[B63] Ellis JW Chen MH Foster M Validated SNPs for eGFR and their Associations with Albuminuria Hum Mol Genet 2012 in press 10.1093/hmg/dds138 PMC3491918 22492995

[B64] Suhre K Shin SY Petersen AK Human metabolic individuality in biomedical and pharmaceutical research Nature 2011 477 54 60 10.1038/nature10354 21886157 PMC3832838

[B65] Gronwald W Klein MS Zeltner R Detection of autosomal dominant polycystic kidney disease by NMR spectroscopic fingerprinting of urine Kidney Int 2011 79 1244 1253 10.1038/ki.2011.30 21389975

[B66] Hindorff LA JH Hall PN Mehta JP Manolio TA A Catalog of Published Genome-Wide Association Studies 2012 Available at: http://www.genome.gov Accessed February 3

[B67] Gudbjartsson DF Holm H Indridason OS Association of variants at UMOD with chronic kidney disease and kidney stones-role of age and comorbid diseases PLoS Genet 2010 6 e1001039 10.1371/journal.pgen.1001039 20686651 PMC2912386

[B68] Pattaro C Genome-wide assocation and functional follow-up reveals new loci for kidney function PLoS Genet 2012 8 1 13 10.1371/journal.pgen.1002584 PMC3315455 22479191

[B69] Köttgen A Hwang SJ Larson MG Uromodulin levels associate with a common UMOD variant and risk for incident CKD J Am Soc Nephrol 2010 21 337 344 10.1681/ASN.2009070725 19959715 PMC2834540

