# Supplementary material for: Study design of DIACORE (DIAbetes COhoRtE) – a cohort study of patients with diabetes mellitus type 2
Source: BMC Med Genet. 2013 Feb 14;14:25. doi: 10.1186/1471-2350-14-25 (PMC3577512; doi:10.1186/1471-2350-14-25)
Supplement: Additional file 1: Table S1 — Physical examination in the core phenotyping protocol. Table S2. Core laboratory parameters. [file 1471-2350-14-25-S1.doc]

**Supplementary** **Table 1:** Physical examination in the core phenotyping protocol.

| Measurement | **Procedure of measurement** | **Apparatus used** |
| --- | --- | --- |
| Blood pressure | Blood pressure is measured after the patient has been seated for 5 minutes using a standard vital signs monitor combined with a standard or large cuff depending on the arm’s circumference (23-33cm or 31-40cm). Blood pressure is measured at the dominant arm placing the cuff at heart level. Measurements are performed three times every 2 minutes. The last two measurements are recorded. | GE Dinamap Vital Signs Monitor, Model V100. CareScape, Germany |
| Heart rate | Recorded with each reading of the blood pressure using a vital signs monitor. The last pulse measurement recorded. | GE Dinamap Vital Signs Monitor, Model V100. CareScape, Germany |
| Weight | Weight in kilograms is measured and recorded to the nearest 0.1 kilogram with light clothing and without shoes and jackets. | Seca digital portable scale, model 701.  Seca Deutschland, Hamburg, Germany. |
| Height | Height is measured to the nearest 0.1 cm using a wall-mounted stadiometer. Participants are asked to remove their shoes and to stand facing forward with heals together and heals, buttocks and shoulders against the wall with arms hanging loosely by their sides. | Wall-mounted stadiometer up to 210 cm, Model 213.  Seca Deutschland, Hamburg, Germany. |
| Waist and hip circumference | Waist and hip circumference are assessed by trained study personnel following removal of heavy outer clothing. | Flexible tape up to 205 cm, Model 201.  Seca Deutschland, Hamburg, Germany. |

**Supplementary Table 2:** Core laboratory parameters.

| **Biomaterial** | **Parameter** |
| --- | --- |
| Serum | Creatinine, Cystatin C, Urea  Calcium, Phosphate  hsCRP, Albumin  Total cholesterol, Triglycerides, LDL, HDL  Uric acid  Insulin* |
| Whole blood | Full blood count  HbA1c, Glucose |
| Urine | Albumin, Creatinine  Dipstick semiquantitative protein, erythrocytes, leukocytes, nitrite |

* Serum insulin activity is obtained from a thawed serum sample.
